# Parental Reports on Early Autism Behaviors in Their Children with Fragile X Syndrome as a Function of Infant Feeding

**DOI:** 10.3390/nu13082888

**Published:** 2021-08-22

**Authors:** Cara J. Westmark

**Affiliations:** Department of Neurology, Molecular & Environmental Toxicology Center, University of Wisconsin, Madison, WI 53706, USA; westmark@wisc.edu; Tel.: +1-608-262-9730

**Keywords:** autism, breast milk, fragile X syndrome (FXS), infant formula, language, soy-based infant formula

## Abstract

This study evaluates the prevalence of autistic behaviors in fragile X syndrome as a function of infant diet. Retrospective survey data from the *Fragile X Syndrome Nutrition Study*, which included data on infant feeding and caregiver-reported developmental milestones for 190 children with fragile X syndrome enrolled in the Fragile X Online Registry with Accessible Database (FORWARD), were analyzed. Exploratory, sex-specific associations were found linking the use of soy-based infant formula with worse autistic behaviors related to language in females and self-injurious behavior in males. These findings prompt prospective evaluation of the effects of soy-based infant formula on disease comorbidities in fragile X syndrome, a rare disorder for which newborn screening could be implemented if there was an intervention. Gastrointestinal problems were the most common reason cited for switching to soy-based infant formula. Thus, these findings also support the study of early gastrointestinal problems in fragile X syndrome, which may underly the development and severity of disease comorbidities. In conjunction with comorbidity data from the previous analyses of the *Fragile X Syndrome Nutrition Study*, the findings indicate that premutation fragile X mothers should be encouraged to breastfeed.

## 1. Introduction

Autism is a complex neurological disorder with core features of impaired communication and social interaction and repetitive stereotypical behavior [1]. Recent prevalence estimates indicate that 1 in 54 children in the United States have an autism spectrum disorder (ASD), with rates 4.3-fold higher in males compared to females [2]. The global prevalence of autism is in the range of 0.8–93 per 1000 [3]. In the majority of cases, the underlying cause of the ASD is not known; however, genetic as well as environmental factors are suspected to contribute to the development and severity of autism [4,5,6,7]. For example, FXS is the leading known genetic cause of autism, with half of males and approximately 20% of females with FXS meeting autism diagnostic criteria and accounting for 5% of autism cases [8,9,10]. There is a dearth of studies assessing the effects of environment on ASD and FXS phenotypes, albeit accumulating evidence suggests that postnatal diet is associated with seizure and autistic behavioral outcomes in autism and FXS models.

Single-source soy-based diets are associated with increased sensitivity to audiogenic-induced seizures in *Fmr1^KO^* mice [11]; increased prevalence of febrile seizures, simple partial seizures and epilepsy comorbidity in autism [12]; deficits in language, communication, social overtures and hypersensitivity to environmental stimuli in autistic children [13]; and increased comorbidity of autism, gastrointestinal problems and allergies in FXS [14]. Conversely, long-term infant feeding with breast milk is associated with decreased prevalence of autism in FXS [15]. There are several potential bioactive components in soy that could reduce seizure threshold and exacerbate autistic behaviors, i.e., soy phytoestrogens and agrochemicals [16,17].

The aim of this study was to begin to fill an important gap in the literature regarding the potential effects of early-life feeding on autistic behavioral outcomes in FXS. Retrospective survey data from the *Fragile X Syndrome Nutrition Study*, which included data on infant feeding and caregiver-reported developmental milestones and autistic behaviors for 190 children with fragile X syndrome enrolled in the Fragile X Online Registry with Accessible Database (FORWARD), were analyzed. FORWARD is a longitudinal database that collects clinician- and parent-reported data on individuals diagnosed with FXS [18]. Exploratory, sex-specific associations were found linking the use of soy-based infant formula with worse autistic behaviors related to language in females and self-injurious behavior in males. This is important considering the dearth of prior diet studies in FXS and the growing consensus that 40–50% of ASD variance could be due to environmental factors including nutrition [7].

## 2. Methods and Subjects

Study Design: The impact of early infant diet on the severity of common FXS phenotypes was assessed through a retrospective survey study utilizing the FORWARD as a sampling frame. The study design, *Fragile X Syndrome Nutrition Study* questionnaire, study population, recruitment success, and data collection have been previously described [14,15]. Data collection for the survey began in November of 2019 and lasted 3 months. The hypothesis explored herein is that early infant diet is associated with specific autistic behaviors in FXS.

Sample Selection and Demographic Characteristics: Inclusion criteria for participation were full-mutation FXS status, caregiver willingness to participate in the study, and completion of behavior-related questions (Q)40, Q41 and Q43–46 on the survey. The dataset included 190 participants who were 74% male and predominantly white (Table 1). The average age of participants on the date of the survey was 18 years (SEM = 0.74). Specific data on socioeconomic status (SES) and educational attainment were not collected.

Ethics Approvals: This study was reviewed by the University of Wisconsin-Madison Health Sciences IRB and determined to meet the criteria for exempt Human Subjects research in accordance with Category 2 defined under 45 CFR-46. Participants provided written informed consent through their enrollment in FORWARD.

Data Collection: Data related to normal child development and autistic behaviors were collected on Q40, Q41 and Q43–Q46 of the *Fragile X Syndrome Nutrition Study* questionnaire (Table 2). Questions were adapted from several standardized instruments including cognitive tools such as the Ages and Stages Questionnaire (ASQ) for 36-months as well as autistic tools such as the Modified Checklist for Autism in Toddlers-Revised (M-CHAT-R) and the Social Responsiveness Scale (SRS) parental survey. Standardized tests were not used due to copyright issues and the inability to make modifications to the questions, for example, verb tense for a retrospective survey. Caregivers were asked to rank the behavior of their child with FXS in seven categories (Language, Cognitive, Child Play, Motor Skills, Autistic Behavior, Hypersensory, and Parent Thought there was a Problem) on a 5-point Likert scale (never, rarely, sometimes, very often, and extremely often), and responses were converted to numerical values (1–5, respectively). Caregivers were instructed to remember how their child behaved when he or she was approximately 3 years old and were told that they could skip any of the questions or the entire section if they did not remember.

Data Analysis: Data were analyzed in accordance with STROBE guidelines. Percentages, means, standard error of the means (SEM) and Student *t*-tests were computed to describe the cohorts. Two-way ANOVA with a Tukey multiple comparison correction was applied to the analysis of individual questions and behavior categories (grouped questions) to examine the null hypotheses that the severity of autistic behaviors in FXS is the same in infants regardless of diet using Prism version 9.0.1 (128) (GraphPad Software, LLC, San Diego, CA, USA). Statistical significance was defined as * *p* < 0.05, ** *p* < 0.01, *** *p* < 0.001, and **** *p* < 0.0001.

## 3. Results

### 3.1. Study Population

The study population included full-mutation FXS males and females, with an average age of 18 years at the time of the survey (Table 1). Caregivers retrospectively answered survey questions regarding disease comorbidities, infant diet and autistic behaviors. Of relevance to this analysis, multi-part questions from survey questions Q40, Q41 and Q43–Q46, which elicited Likert-scale responses regarding normal child development and the severity of autistic phenotypes, were posed to caregivers. Considering the average age at the time of the survey (18 years old) and the retrospective age of the queries (3 years old), there was a high response rate of 98% or greater for each question related to child development or autistic behavior despite caregivers being instructed to skip questions that they did not remember (Table 2). Only 9 out of 199 caregivers skipped the entire section of behavior questions and 13 skipped 1–3 individual questions. Two of the nine participants with blank answers to all of the behavior questions were adopted. Thus, there was an overall high response rate to questions on early childhood behavior indicating confidence on the part on the respondents regarding recall.

### 3.2. Autism Behavior Scores

A total FXS toddler behavior score was calculated by subtracting the point total for caregiver-reported autistic-related behaviors (Q43c + Q44a + Q44b +Q44c + Q44d + Q44e + Q44f + Q45a + Q45b + Q45c + Q45d + Q46a + Q46b + Q46c + Q46d) from the point total for typical childhood behaviors (Q40a + Q40b + Q40c + Q40d + Q40e + Q41a + Q41b + Q41c + Q41d + Q41e + Q41f + Q43a + Q43b + Q43d + Q43e + Q43f + Q45e). This calculated behavior score based on 32 behaviors for participants whose caregivers reported an answer of “No” to Q1a: “*Does your child with* *Fragile X Syndrome also have Autism?*” was 15 (SEM 2.0, N = 83) compared to those who answered “Yes” to Q1a having an average score of −2.1 (SEM 1.7, N = 83) with a *p* value = 7.8 ^x^ 10^-10^ by Student *t*-test. Thus, caregiver-reported incidence of autism (Q1a) strongly agreed with reported Likert-scale scores for autistic behaviors in their children with FXS.

The behavior questions were binned into categories based on relevance to “Language” (Q40a, Q40b, Q40c, Q40d, Q40e), “Cognitive” (Q41a, Q41b, Q41c, Q41d, Q41e, Q41f), “Child Play” (Q43a, Q45e), “Motor Skills” (Q43b, Q43d, Q43e, Q43f), “Autistic Behaviors” (Q44b, Q44f, Q43c), “Hypersensory” (Q44a, Q44c, Q44d, Q44e, Q45a, Q45b, Q45c, Q45d) and “Parent Thought Problem” (Q46a, Q46b, Q46c, Q46d) and analyzed by two-way ANOVA as a function of sex and diet. First, the data were analyzed as a function of breast milk consumption (Q18) and then as a function of soy-infant formula consumption (Q28). For the breast milk cohorts [breast milk (n = 33 female and 102 male) versus no breast milk (n = 17 female and 35 male)], sex-specific differences were found in Language, Cognition, Child Play, Motor Skills, and Autistic Behaviors, but not in Hypersensory or Parent Thought Problem categories (Table 3, Supplementary Figure S1, Supplementary Table S1). Multiple comparisons analyses found higher language and lower autistic behavior scores in females compared to males with breast milk feeding, and higher cognition scores in females compared to males with formula feeding (Table 3). No diet-based differences (breast versus no breast milk) were found with grouped or individual questions (Table 3, Supplementary Figures S1–S8, Supplementary Table S1). The total behavior score was 3.4-fold higher in females than males fed breast milk, with trends for increased scores in both females and males fed breast milk compared to no breast milk (Table 3, Supplementary Figure S1, Supplementary Table S1).

Analysis of subjects exclusively fed breast milk for 12 months or longer (i.e., no reporting of any formula use, n = 14 females and 41 males) indicated sex-specific differences in Language, Cognition, Motor Skills, and Autistic Behaviors and diet-specific differences in Language, Autistic Behaviors, and Hypersensory compared to the no breast milk cohort (n = 17 females and 35 males) (Supplementary Table S2). Multiple comparison analysis confirmed higher language scores in females versus males fed breast milk and higher cognitive scores in females versus males with formula feeding, but found no significant differences as a function of diet (Supplementary Figure S9).

For the no-soy versus soy-based infant formula cohorts, data were analyzed by two-way ANOVA based on sex and diet [soy-based infant formula (n = 10 female and 32 male) versus no soy (n = 36 female and 92 male)] with sex-specific differences found in Language, Cognition, Motor Skills, and Autistic Behaviors, but not in the Child Play, Hypersensory or Parent Thought Problem categories (Table 4, Supplementary Figure S10). Soy diet-specific differences were observed for Language, Autistic Behaviors, and Parent Thought Problem (Supplementary Table S3). Multiple comparisons analyses indicated a significantly higher language category score in females not fed soy-based infant formula compared to all other groups as well as higher cognition and motor skills scores and decreased autistic behaviors in females versus males not fed soy-based infant formula (Table 4). The autistic behaviors score was the highest in males fed soy-based infant formula and significantly reduced in males not fed soy. The total behavior score was 3.3-fold higher in females than males in the no soy cohort with trends for decreased scores in both females and males fed soy compared to no soy (Table 4, Supplementary Figure S10, Supplementary Table S3).

### 3.3. Indiviudal Autistic Behaviors

Analysis of individual questions related to Language indicated a 1.5-fold decrease in Q40a (talk), a 1.7-fold decrease in Q40d (three word sentences), and a 1.6-fold decrease in Q40e (use words to request things) in females such that females reporting the use of soy-based infant formula exhibited decreased expressive language scores that matched the language scores of males on either non-soy or soy-based infant formula (Table 4, Supplementary Figure S11). Analysis of individual questions for the Cognition category indicated a 1.2-fold decrease in Q41a (correctly ID) and a 1.5-fold decrease in Q41e (play pretend) in males compared to females not fed soy with the sex-specific differences lost with soy-based infant formula (Table 4, Supplementary Figure S12). Analysis of individual questions for the Autism Behaviors category indicated a 1.6-fold increase in Q44b (rocking/hand flapping/spinning) when comparing females to males not fed soy, and the sex-specific difference was lost with soy-based infant formula (Table 4, Supplementary Figure S13). There was a 1.4-fold increase in Q44f (injure self) for males fed soy-based infant formula. Analyses of individual questions for the Child Play, Motor Skills and Hypersensory categories indicated no significant differences as a function of soy-based infant formula for boys or girls with FXS (Supplementary Figures S14–S16). There was a sex-specific 1.3-fold decrease in Q43f (dress self) in males compared to females not fed soy (Table 4, Supplementary Figure S15). Analysis of individual questions related to Parent Thought Problem indicated a 1.2-fold increase in Q46d (learning problem) in females fed soy and when comparing females to males not fed soy (Table 4, Supplementary Figure S17).

There were a small number of subjects reporting use of a single diet [only breast milk (n = 8 female and 32 male), cow milk-based formula (n = 7 female and 9 male) or soy-based formula (n = 2 female and 4 male)]. Analysis as a function of single diets indicated an interaction between sex and diet for Motor Skills but no other sex- or diet-specific differences with the small cohort sizes (Supplementary Figure S18, Supplementary Table S4).

## 4. Discussion

There is a paucity of studies examining the effects of diet in FXS [11,14,15,19,20,21,22,23,24]. Herein, diet-responsive autistic behaviors in FXS included three behaviors in girls within the Language category (talk, speak in 3-word sentences and use words to request things), one behavior in girls within the Parent Thought Problem category (learning problem), and one behavior in boys within the Autistic Behaviors category (injure self) in association with soy-based infant formula. Comparison of these exploratory autistic phenotypes in FXS, which are all worse in association with soy-based infant formula, with an earlier retrospective analysis in subjects with autism from the Simons Foundation Autism Research Initiative (SFARI) indicate overlap in the areas of language in females and self-injurious behavior in males. Specifically, sub-scale 5 of the Aberrant Behavior Checklist (ABC), Inappropriate Speech, is worse in SFARI females fed soy-based infant formula. There is a statistically significant increase in the Autism Diagnostic Interview-Revised (ADI-R) Total Restricted, Repetitive and Stereotyped Behavior (RRSB) score in SFARI males. There is also a 1.3-fold increase in self-injurious behavior in SFARI males fed soy-based infant formula as evidenced by line-item question #83 of the ADI-R [13]. Overall, these data suggest that expressive language in females and more severe self-injurious behavior in males are associated with a soy-enriched diet early in life.

Sex-specific differences in autism outcomes in FXS are not unexpected. ASD is far more prevalent in males. Females with FXS demonstrate better receptive and expressive vocabulary than boys with FXS [25,26]. Males with comorbid FXS and autism exhibit worse communication, social, functional academic and daily living skills, increased behavior problems, and greater cognitive disability [27,28,29,30,31,32,33,34,35]. Self-injurious behavior is a common phenotype in FXS, affecting 31–79% of males but only 10–17% of females, with onset occurring within the first 3 years of age for 60% of subjects [36,37].

Expressive language sampling is under development as an outcome measure for FXS clinical trials [38,39]. Infants and toddlers with FXS exhibit deficits in early language skills compared to typically developing peers [40,41,42]. Children with FXS and comorbid autism are more impaired in nonverbal cognition and expressive language while receptive language is a relative strength for children with FXS (without autism) [43]. Furthermore, expressive language is associated with quantitative reasoning whereas receptive language is associated with adaptive behavior in girls with FXS [44]. Our findings suggest that expressive language in females can is associated with early-life diet.

The methylation silencing and mosaicism status of the *FMR1* gene in the FXS participants in this study are not known. DNA methylation status of the CGG repeats in the *FMR1* gene promoter at birth is predictive of later intellectual function and autism features [45]. The majority of males with FXS express some *FMR1* mRNA and this incomplete silencing of the promoter is associated with increased autistic features [46,47,48,49], In males, full mutation FXS with complete or incomplete methylation silencing of the *FMR1* gene is associated with increased inappropriate speech on the ABC-C sub-scale compared to the FXS group mosaic for pre- and full mutation alleles, and mosaicism in either sex is associated with less maladaptive behaviors [50,51]. These data suggest that comorbid autism phenotypes in FXS could be affected by gene–environmental interactions that regulate *FMR1* methylation and/or expression.

At this time, soy bioactive component(s) that are associated with worse autism behaviors have not been identified. Potential candidates include endogenous phytoestrogens and contaminating agrochemicals. The soy phytoestrogen daidzein is associated with increased seizure activity in mice [11], and the widely used herbicide glyphosate and its metabolite aminomethylphosphonic acid (AMPA) are found in soy-based infant formulas [52]. Increased severity and frequency of disease symptoms were found in FXS premutation carriers in the agricultural community of Ricaurte, Colombia and hypothesized to be associated with higher exposure to neurotoxic pesticides [53]. It remains to be determined if a bioactive component of soy or an altered amino acid profile contributes to an association with more severe autistic behaviors.

The limitations of this study include the small study size, the data are retrospective and dependent on parental recall without clinical documentation (average age of participants was 18 years), this study was not powered to detect multiple hypotheses, there were no age-matched control data in typically developing children, and standardized autism diagnostic tests were not used. However, several standardized instruments were used to inform the survey questions, including cognitive tools such as the Ages and Stages Questionnaire (ASQ) for 36-months as well as autistic tools such as the Modified Checklist for Autism in Toddlers-Revised (M-CHAT-R) and the Social Responsiveness Scale (SRS) parental survey. Despite these limitations, several exploratory autistic behaviors were identified that are associated with the consumption of soy-based infant formula. Of particular interest is the association between soy-based infant formula and worse language scores in the female participants with FXS. A possible criticism of this work is that children could have been switched from breast milk to infant formula for various diagnosed or undiagnosed health issues that predisposed them to develop more severe autistic behaviors. A prospective study will be required to help address these confounding issues.

The strengths of the study design include the study is the first to link caregiver-reported data on infant feeding practices in children with FXS to specific developmental and autistic behaviors in those children; FORWARD offers a unique study population of subjects with established FXS; and the caregivers are a highly motivated group of parents eager to participate in research studies.

If early-life nutrition provides a practical intervention to reduce the severity of autism phenotypes in FXS, it would be imperative to implement newborn screening (NBS) protocols for the early identification of infants with FXS that could benefit from nutritional management. Currently, genetic testing for FXS is not included in the NBS panel because there are no proven medical treatments [54]. Thus, the majority of children with FXS are not identified until at least 3 years of age [55]. The human brain is highly plastic during the first two years of life, suggesting that earlier intervention would be more therapeutic. Voluntary NBS for FXS indicates that only 62% of parents agree to have their child screened, suggesting a need for stronger evidence of benefit before implementation of a nationwide screening program [56].

## 5. Conclusions

In conclusion, the investigation of dietary effects on FXS phenotypes is an understudied field with significant potential to provide a safe, easily implemented intervention to reduce GI problems, autism and other disease comorbidities in FXS. The retrospective survey findings reported herein suggest that the consumption of soy-based infant formula is associated with deficits in language, repetitive behavior and self-injurious behavior in FXS. In conjunction with the previous analyses of the questionnaire, the findings indicate that breastmilk is associated with improved outcomes in FXS compared to soy-based infant formula and that mothers should be encouraged to breastfeed. It remains to be determined if soy-based infant formula use is a cause or consequence of FXS comorbidities. Future research directions should include increasing the sample size and ethnic diversity of the *Fragile X Syndrome Nutrition Study* as well as prospective evaluation of comorbidities in participants with FXS as a function of diet. The findings have relevance to all ASD, which are estimated at 52 million cases, or 1–2% of children, worldwide [57]. Early gastrointestinal dysfunction is an endophenotype of ASD that is mitigated with breast milk [58]. It remains to be determined if soy-based infant formula exacerbates gastrointestinal problems in ASD.

## Figures and Tables

**Table 1:** Demographics.

Race/Ethnicity	Female (N = 50)	Male (N = 140)
American Indian or Alaskan Native (%)	0	0.71
Asian (%)	2	1.4
Black or African American (%)	0	5
Hispanic or Latino (%)	8	5
Native Hawaiian or Other Pacific Islander (%)	0	0
White (%)	92	94
Other Race or Ethnicity (%)	2	0
Average Age at Time of Survey (Years, SEM)	18 (1.45)	18 (0.87)

**Table 2:** Assessed Behaviors.

Question: How Often Did Your Child…	N	Class *
Language		
Q40a…talk?	187	T
Q40b…say his or her name when asked?	189	T
Q40c…respond when spoken to, for example, did your child look at you when you called his or her name?	190	T
Q40d…speak in sentences of at least 3 words?	190	T
Q40e…use words to request things, for example when he or she wanted a cookie?	189	T
Cognition		
Q41a…correctly identify people or objects when you pointed to them and asked what they were, for example, “mommy”, “daddy”, “dog”, or “airplane”?	187	T
Q41b…follow simple directions, for example “sit down” or “get your shoes”?	190	T
Q41c…point at things to request them, for example, a toy on a shelf?	190	T
Q41d…copy others, for example, clapping their hands or waving?	189	T
Q41e…play pretend, for example, rocking a doll to sleep or feeding a stuffed animal?	189	T
Q41f…have savant ability, a restricted skill superior to their age group, for example reading early, or memorizing books?	190	T
Child Play		
Q43a…like motion activities, for example, to be swung or bounced?	189	T
Q45e…like to play with other children?	190	T
Motor Skills		
Q43b…walk?	188	T
Q43d…pick up small objects, for example, Cheerios?	189	T
Q43e…feed him or herself with a spoon?	189	T
Q43f…help dress him or herself, for example pull up their pants?	190	T
Autistic Behaviors		
Q44b…do rocking, hand flapping or spinning over and over again?	190	A
Q44f…try to injure him or herself, for example, head banging?	190	A
Q43c…toe walk?	187	A
Hypersensory		
Q44a…get upset by loud noises, for example, the vacuum cleaner or microwave?	189	A
Q44c…cry excessively over small hurts?	190	A
Q44d…have temper outbursts if he or she did not get their way?	190	A
Q44e…isolate him or herself?	189	A
Q45a…get upset by minor changes to their daily routine?	190	A
Q45b…have difficulty expressing his or her needs and desires?	190	A
Q45c…hate crowds, for example, difficulties in restaurants or the grocery store?	190	A
Q45d…not like to be touched or held?	190	A
Parent Thought Problem		
Q46a…had an anxiety problem?	190	A
Q46b…had a hearing problem?	190	A
Q46c…had a vision problem?	190	A
Q46d…had a learning problem?	190	A

* Behaviors from Q40, Q41 and Q43–Q46 of the *Fragile X Syndrome Nutrition Study questionnaire* were classified as typical (T) or autistic (A). N is the number of respondents who answered the question by ranking their child’s behavior on a 5-point Likert scale (never, rarely, sometimes, very often, and extremely often).

**Table 3:** Caregiver-reported autism phenotypes as a function of breast milk.

Category	Female No BM *	Female Plus BM	Male No BM	Male Plus BM	*p* ^#^
Language (average, SEM, N)					
Q40a (talk)	3.00, 0.26, 17	3.28, 0.25, 32	2.23, 0.18, 35	2.58, 0.11, 100	d,e
Q40b (say name)	2.82, 0.30, 17	3.12, 0.27, 33	2.00, 0.18, 35	2.27, 0.12, 101	d,e
Q40c (respond)	3.82, 0.26, 17	4.18, 0.13, 33	3.31, 0.17, 35	3.68, 0.10, 102	d
Q40d (3 word sentences)	2.65, 0.34, 17	2.73, 0.27, 33	1.74, 0.17, 35	1.95, 0.12, 102	d,e
Q40e (words to request things)	2.69, 0.33, 16	3.09, 0.26, 33	2.17, 0.19, 35	2.44, 0.12, 102	d
Language Sub-Score	3.00, 0.23, 17	3.27, 0.22, 33	2.29, 0.14, 35	2.58, 0.09, 102	d,e
Cognition (average, SEM, N)					
Q41a (correctly identify)	3.38, 0.29, 16	3.47, 0.25, 32	2.77, 0.20, 35	2.98, 0.12, 101	-
Q41b (follow directions)	3.29, 0.25, 17	3.52, 0.19, 33	2.71, 0.15, 35	2.90, 0.11, 102	d,e
Q41c (point to request)	3.82, 0.23, 17	3.52, 0.21, 33	3.14, 0.18, 35	3.08, 0.12, 102	-
Q41d (copy others)	3.65, 0.21, 17	3.42, 0.20, 33	2.77, 0.19, 35	3.16, 0.11, 101	b
Q41e (play pretend)	2.88, 0.31, 17	2.63, 0.25, 32	1.71, 0.19, 35	1.96, 0.12, 102	b,c,d,e
Q41f (have savant ability)	1.71, 0.33, 17	1.27, 0.13, 33	1.20, 0.09, 35	1.30, 0.08, 102	-
Cognitive Sub-Score	3.11, 0.19, 17	2.97, 0.15, 33	2.39, 0.12, 35	2.56, 0.08, 102	b,c,d
Child Play (average, SEM, N)					
Q43a (likes motion)	4.29, 0.19, 17	4.00, 0.19, 33	3.97, 0.16, 34	4.12, 0.10, 102	-
Q45e (like to play with other kids)	3.00, 0.28, 17	3.15, 0.17, 33	2.40, 0.17, 35	2.88, 0.12, 102	d
Child Play Sub-Score	3.65, 0.018, 17	3.58, 0.13, 33	3.14, 0.12, 35	3.50, 0.08, 102	-
Motor Skills (average, SEM, N)					
Q43b (walk)	4.13, 0.22, 16	4.39, 0.14, 33	3.85, 0.19, 34	4.21, 0.08, 102	-
Q43d (picks up small objects)	4.00, 0.27, 17	4.09, 0.19, 32	3.51, 0.18, 35	3.54, 0.10, 102	e
Q43e (feeds self with spoon)	3.53, 0.29, 17	3.55, 0.23, 33	2.69, 0.19, 35	3.06, 0.13, 101	d
Q43f (help dress self)	3.24, 0.34, 17	3.12, 0.23, 33	2.26, 0.18, 35	2.61, 0.12, 102	b,d
Motor Skills Sub-Score	3.72, 0.23, 17	3.79, 0.16, 33	3.07, 0.15, 35	3.35, 0.08, 102	d
Autistic Behaviors (average, SEM, N)					
Q44b (rocking, hand flapping)	3.06, 0.39, 17	2.36, 0.26, 33	3.71, 0.24, 35	3.74, 0.13, 102	d,e
Q44f (injure self)	1.53, 0.21, 17	1.48, 0.17, 33	1.77, 0.21, 35	2.06, 0.14, 102	-
Q43c (toe walk)	2.41, 0.42, 17	2.30, 0.24, 33	2.38, 0.23, 32	2.05, 0.12, 102	-
Autistic Behavior Sub-Score	2.33, 0.23, 17	2.05, 0.17, 33	2.67, 0.17, 35	2.61, 0.09, 102	d,e
Hypersensory (average, SEM, N)					
Q44a (upset by loud noises)	3.71, 0.28, 17	3.00, 0.22, 33	3.21, 0.21, 34	3.17, 0.13, 102	-
Q44c (cry excessively)	2.88, 0.32, 17	2.61, 0.23, 33	2.46, 0.19, 35	2.26, 0.11, 102	-
Q44d (temper outbursts)	3.12, 0.30, 17	3.06, 0.21, 33	2.86, 0.19, 35	2.97, 0.12, 102	-
Q44e (isolate self)	2.94, 0.35, 17	2.09, 0.16, 33	2.51, 0.19, 35	2.56, 0.12, 101	-
Q45a (upset by minor changes)	3.41, 0.26, 17	3.09, 0.18, 33	3.34, 0.17, 35	3.31, 0.11, 102	-
Q45b (difficulty expressing needs)	3.82, 0.20, 17	3.70, 0.20, 33	4.23, 0.14, 35	4.03, 0.09, 102	-
Q45c (hate crowds)	3.35, 0.28, 17	3.12, 0.23, 33	3.57, 0.17, 35	3.47, 0.13, 102	-
Q45d (not liked to be touched)	2.47, 0.26, 17	2.12, 0.18, 33	2.54, 0.19, 35	2.39, 0.12, 102	-
Hypersensory Sub-Score	3.21, 0.18, 17	2.85, 0.11, 33	3.09, 0.11, 35	3.02, 0.07, 102	-
Parent Thought Problem (average, SEM, N)					
Q46a (anxiety problem)	3.53, 0.31, 17	3.24, 0.24, 33	3.51, 0.24, 35	3.41, 0.13, 102	-
Q46b (hearing problem)	1.76, 0.29, 17	1.55, 0.17, 33	1.74, 0.19, 35	1.83, 0.12, 102	-
Q46c (vision problem)	1.47, 0.27, 17	1.76, 0.21, 33	1.71, 0.20, 35	1.69, 0.12, 102	-
Q46d (learning problem)	4.18, 0.30, 17	4.03, 0.22, 33	4.60, 0.14, 35	4.47, 0.08, 102	-
Parent Thought Problem Sub-Score	3.10, 0.14, 17	2.92, 0.07, 33	2.95, 0.07, 35	3.01, 0.04, 102	-
Total Behavior Score (average, SEM, N)	9.53, 4.39, 15	16.57, 3.49, 30	−0.31, 2.64, 26	4.84, 1.95, 92	d,e

* No BM = no breast milk, Plus BM = fed breast milk. ^#^ Statistically significant results by Tukey’s multiple comparison test are denoted: (a) female no BM versus female plus BM, (b) female no BM versus male no BM, (c) female no BM versus male plus BM, (d) female plus BM versus male no BM, (e) female plus BM versus male plus BM, and (f) male no BM versus male plus BM.

**Table 4:** Caregiver-reported autism phenotypes as a function of soy-based infant formula.

Category	Female No Soy *	Female Plus Soy	Male No Soy	Male Plus Soy	*p* ^#^
Language (average, SEM, N)					
Q40a (talk)	3.44, 0.20, 36	2.22, 0.22, 9	2.51, 0.11, 91	2.35, 0.23, 31	a,b,c
Q40b (say name)	3.22, 0.24, 36	2.30, 0.37, 10	2.22, 0.12, 91	2.03, 0.22, 32	b,c
Q40c (respond)	4.06, 0.13, 36	3.90, 0.38, 10	3.58, 0.11, 92	3.53, 0.20. 32	-
Q40d (3 word sentences)	2.94, 0.25, 36	1.70, 0.21, 10	1.89, 0.11, 92	1.75, 0.22, 32	a,b,c
Q40e (words to request things)	3.23, 0.23, 35	2.00, 0.21, 10	2.34, 0.11, 92	2.25, 0.23, 32	a,b,c
Language Sub-Score	3.38, 0.18, 36	2.42, 0.17, 10	2.50, 0.09, 92	2.38, 0.18, 32	a,b,c
Cognition (average, SEM, N)					
Q41a (correctly identify)	3.57, 0.22, 35	2.90, 0.38, 10	2.91, 0.12, 91	2.81, 0.26, 32	b
Q41b (follow directions)	3.31, 0.18, 36	3.40, 0.27, 10	2.87. 0.11, 92	2.72, 0.19, 32	-
Q41c (point to request)	3.56, 0.17, 36	3.70, 0.42, 10	3.03, 0.12, 92	2.97, 0.24, 32	-
Q41d (copy others)	3.47, 0.16, 36	3.30, 0.42, 10	3.00, 0.12, 91	3.09, 0.21, 32	-
Q41e (play pretend)	2.74, 0.23, 35	2.40, 0.43, 10	1.79, 0.11, 92	1.91, 0.24, 32	b,c
Q41f (have savant ability)	1.39, 0.18, 36	1.50, 0.27, 10	1.23, 0.07, 92	1.41, 0.17, 32	-
Cognitive Sub-Score	3.00, 0.14, 36	2.87, 0.24, 10	2.47, 0.08, 92	2.48, 0.17, 32	b,c
Child Play (average, SEM, N)					
Q43a (likes motion)	4.11, 0.16, 36	4.40, 0.22, 10	3.98, 0.11, 92	4.29, 0.16, 31	-
Q45e (like to play with other kids)	3.19, 0.18, 36	2.90, 0.28, 10	2.72, 0.13, 92	2.78, 0.19, 32	-
Child Play Sub-Score	3.65, 0.13, 36	3.65, 0.15, 10	3.35, 0.09, 92	3.48, 0.14, 32	-
Motor Skills (average, SEM, N)					
Q43b (walk)	4.31, 0.14, 36	4.22, 0.28, 9	4.14, 0.09, 92	4.10, 0.16, 31	-
Q43d (picks up small objects)	3.94, 0.20, 35	4.20, 0.25, 10	3.52, 0.10, 92	3.53, 0.18, 32	-
Q43e (feeds self with spoon)	3.61, 0.20, 36	3.30, 0.42, 10	3.01, 0.13, 91	2.78, 0.22, 32	c
Q43f (help dress self)	3.25, 0.22, 36	3.10, 0.41, 10	2.54, 0.11, 92	2.28, 0.25, 32	b,c
Motor Skills Sub-Score	3.78, 0.16, 36	3.69, 0.21, 10	3.30, 0.08, 92	3.16, 0.16, 32	b,c
Autistic Behaviors (average, SEM, N)					
Q44b (rocking, hand flapping)	2.28, 0.24, 36	3.50, 0.48, 10	3.55, 0.14, 92	4.16, 0.23, 32	b,c
Q44f (injure self)	1.39, 0.13, 36	1.90, 0.43, 10	1.77, 0.13, 92	2.47, 0.28, 32	c,f
Q43c (toe walk)	2.36, 0.25, 36	2.20, 0.47, 10	2.12, 0.13, 91	2.42, 0.27, 31	-
Autistic Behavior Sub-Score	2.01, 0.15, 36	2.53, 0.39, 10	2.48, 0.08, 92	3.04, 0.17, 32	b,c,f
Hypersensory (average, SEM, N)					
Q44a (upset by loud noises)	3.22, 0.19, 36	3.60, 0.48, 10	2.96, 0.13, 91	3.41, 0.25, 32	-
Q44c (cry excessively)	2.78, 0.20, 36	2.30, 0.50, 10	2.27, 0.12, 92	2.34, 0.23, 32	-
Q44d (temper outbursts)	3.06, 0.20, 36	3.30, 0.45, 10	2.84, 0.12, 92	3.25, 0.22, 32	-
Q44e (isolate self)	2.58, 0.20, 36	2.20, 0.36, 10	2.47, 0.12, 92	2.81, 0.20, 32	-
Q45a (upset by minor changes)	3.17, 0.18, 36	3.40, 0.31, 10	3.28, 0.11, 92	3.41, 0.23, 32	-
Q45b (difficulty expressing needs)	3.61, 0.19, 36	4.10, 0.18, 10	3.99, 0.09, 92	4.38, 0.15, 32	c
Q45c (hate crowds)	3.17, 0.22, 36	3.50, 0.31, 10	3.32, 0.13, 92	3.72, 0.21, 32	-
Q45d (not liked to be touched)	2.00, 0.15, 36	2.70, 0.40, 10	2.32, 0.12, 92	2.47, 0.23, 32	-
Hypersensory Sub-Score	2.95, 0.12, 36	3.14, 0.22, 10	2.92, 0.07, 92	3.22, 0.14, 32	-
Parent Thought Problem (average, SEM, N)					
Q46a (anxiety problem)	3.25, 0.22, 36	3.60, 0.40, 10	3.38, 0.14, 92	3.34, 0.26, 32	-
Q46b (hearing problem)	1.61, 0.19, 36	1.70, 0.30, 10	1.75, 0.12, 92	1.94, 0.23, 32	-
Q46c (vision problem)	1.67, 0.20, 36	1.50, 0.40, 10	1.48, 0.11, 92	2.06, 0.24, 32	-
Q46d (learning problem)	3.86, 0.23, 36	4.80, 0.13, 10	4.51, 0.08, 92	4.44, 0.18, 32	a,b
Parent Thought Problem Sub-Score	2.94, 0.08, 36	3.12, 0.11, 10	2.92, 0.04, 92	3.12, 0.08, 32	-
Total Behavior Score (average, SEM, N)	16.19, 3.14, 32	5.78, 4.99, 9	4.98. 1.83, 83	0.30, 4.11, 27	b,c

* No Soy = no soy-based infant formula, Plus Soy = fed soy-based infant formula. ^#^ Statistically significant results by Tukey’s multiple comparison test are denoted: (a) female no soy versus female plus soy, (b) female no soy versus male no soy, (c) female no soy versus male plus soy, (d) female plus soy versus male no soy, (e) female plus soy versus male plus soy, and (f) male no soy versus male plus soy.

## Data Availability

Data are contained within the article, Supplementary Material or cited work, or available on request from the corresponding author.

## Supplementary Materials

The following are available online at https://www.mdpi.com/article/10.3390/nu13082888/s1, Figure S1: Average behavior scores as a function of breast milk, Figure S2: Average language scores as a function of breast milk, Figure S3: Average cognition scores as a function of breast milk, Figure S4: Average child play a function of breast milk, Figure S5: Average motor skills as a function of breast milk, Figure S6: Average autism behavior scores as a function of breast milk, Figure S7: Average hypersensory scores as a function of breast milk, Figure S8: Average parent thought there was a problem scores as a function of breast milk, Figure S9: Average behavior scores as a function of breast milk for 12 months, Figure S10: Average behavior scores as a function of soy-based infant formula, Figure S11: Average language scores as a function of soy-based infant formula, Figure S12: Average cognition scores as a function of soy-based infant formula, Figure S13: Average autism behavior scores as a function of soy-based infant formula, Figure S14: Average child play scores as a function of soy-based infant formula, Figure S15: Average motor skills scores as a function of soy-based infant formula, Figure S16: Average hypersensory scores as a function of soy-based infant formula, Figure S17: Average parents thought there was a problem scores as a function of soy-based infant formula, Figure S18: Average behavior scores as a function of single milk, Table S1: Average behavior category statistics as a function of breast milk, Table S2: Average behavior category statistics as a function of breast milk 12 months, Table S3: Average behavior category statistics as a function of soy formula, Table S4: Average behavior category statistics as a function of single diet.
