# Supplementary material for: Parental Reports on Early Autism Behaviors in Their Children with Fragile X Syndrome as a Function of Infant Feeding
Source: Nutrients. 2021 Aug 22;13(8):2888. doi: 10.3390/nu13082888 (PMC8401950; doi:10.3390/nu13082888)
Supplement: Supplementary file 1 [file nutrients-13-02888-s001.zip › nutrients-1315078-SI.pdf]

Supplementary Data

Parental Reports on Early Autism Behaviors in Their Children with Fragile X Syndrome as a Function of Infant Feeding

Cara J. Westmark

Department of Neurology, Molecular & Environmental Toxicology Center, University of Wisconsin, Madison, WI, 53706, USA; westmark@wisc.edu; Tel: +1-608-262-9730

Supplementary Figure S1. Average behavior scores as a function of breast milk. The average behavior scores for grouped Language, Cognition, Child Play, Motor Skills, Autistic Behaviors, Hypersensory, and Parent Though Problem questions as well as the Total behavior score were plotted versus sex where white bars represent no breast milk and blue bars represent plus breast milk. Error bars represent SEM. Asterisks indicate statistical significance by 2-way ANOVA and Tukey posthoc tests as defined as **P*<0.05, ***P*<0.01, and ****P*<0.001.

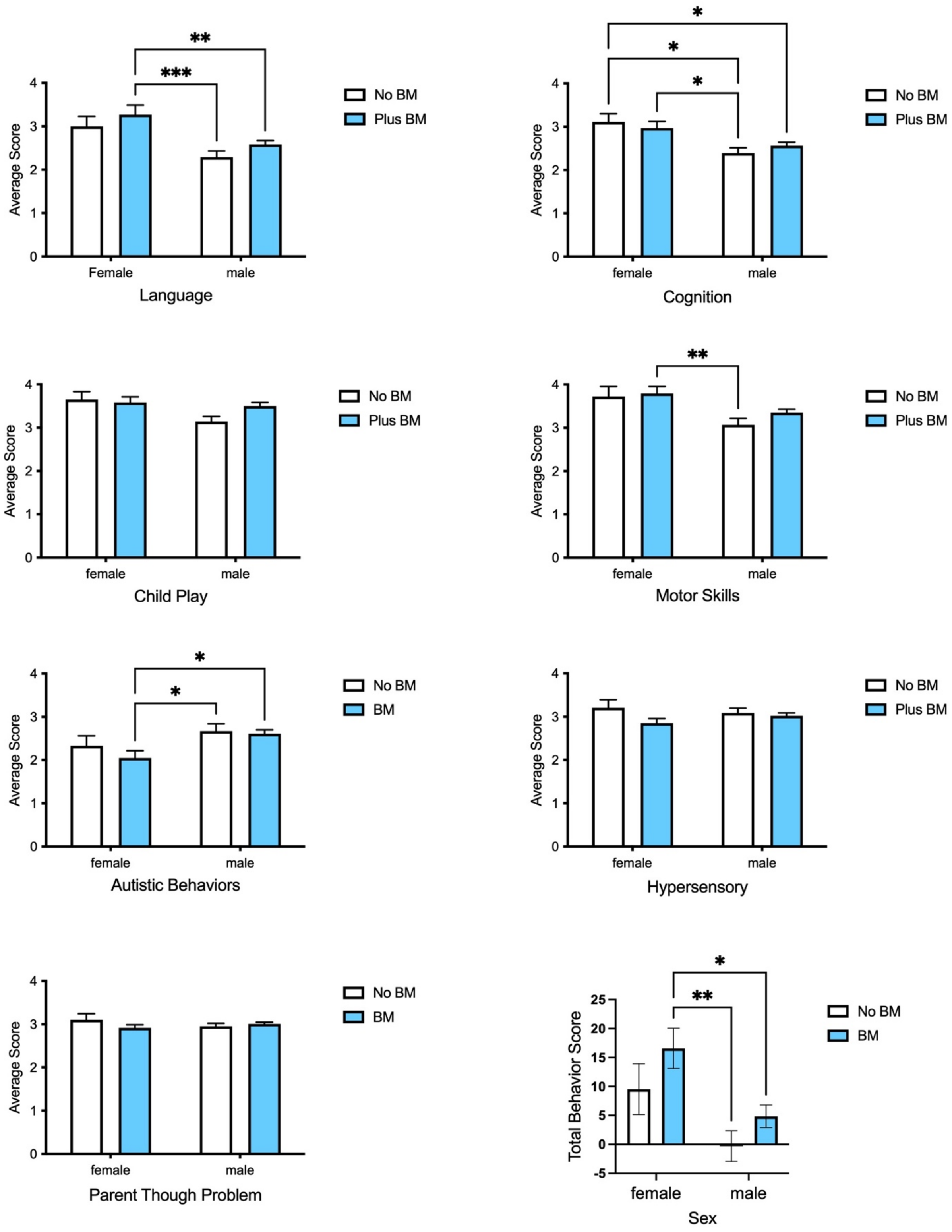

Supplementary Figure S2. Average language scores as a function of breast milk. The average scores for individual Language questions were plotted versus sex where white bars represent no breast milk and blue bars represent plus breast milk. Error bars represent SEM. Asterisks indicate statistical significance by 2-way ANOVA and Tukey posthoc tests as defined as *$P<0.05$ and **$P<0.01$.

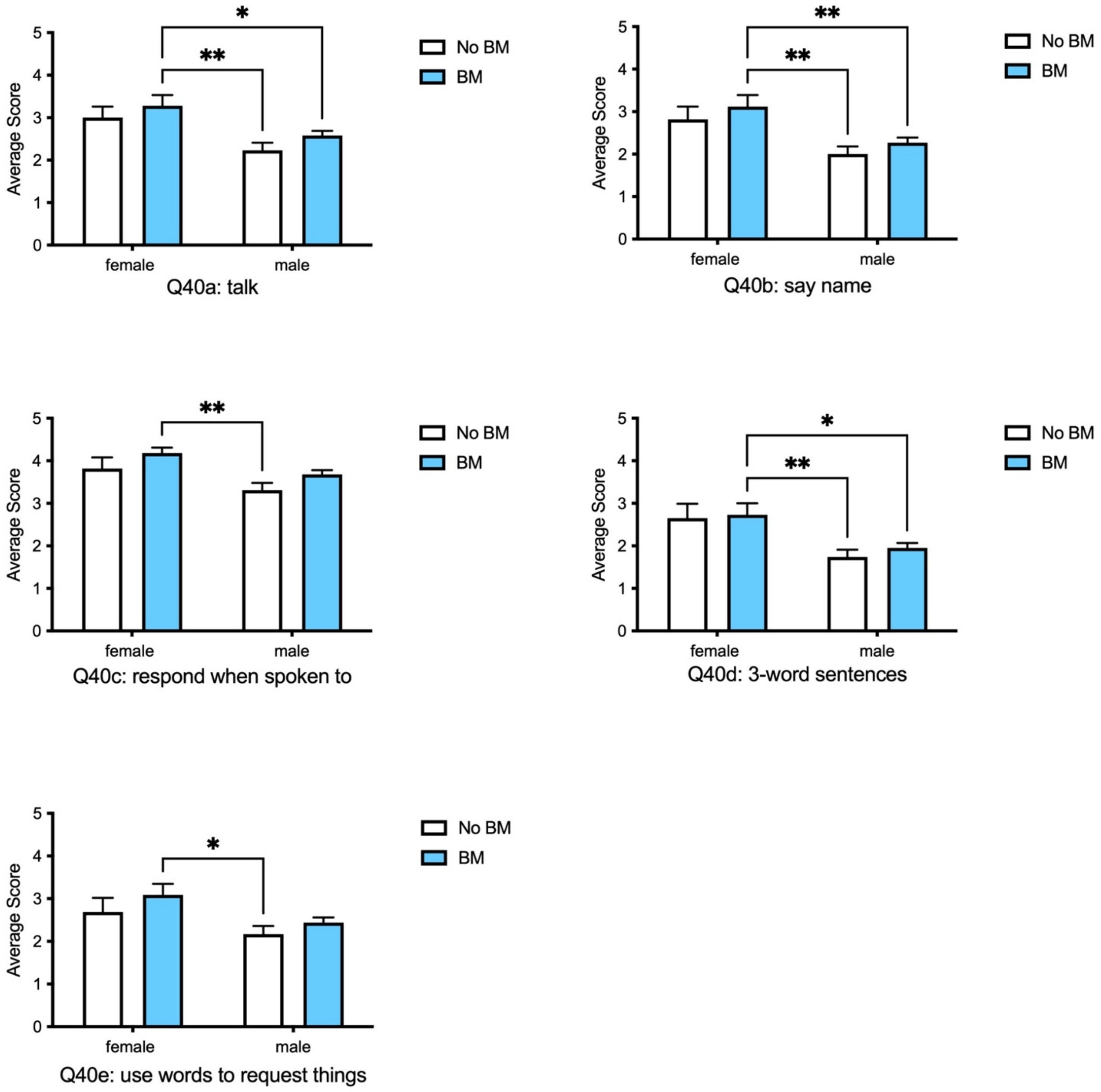

Supplementary Figure S3. Average cognition scores as a function of breast milk. The average scores for individual Cognition questions were plotted versus sex where white bars represent no breast milk and blue bars represent plus breast milk. Error bars represent SEM. Asterisks indicate statistical significance by 2-way ANOVA and Tukey posthoc tests as defined as *P<0.05 and **P<0.01.

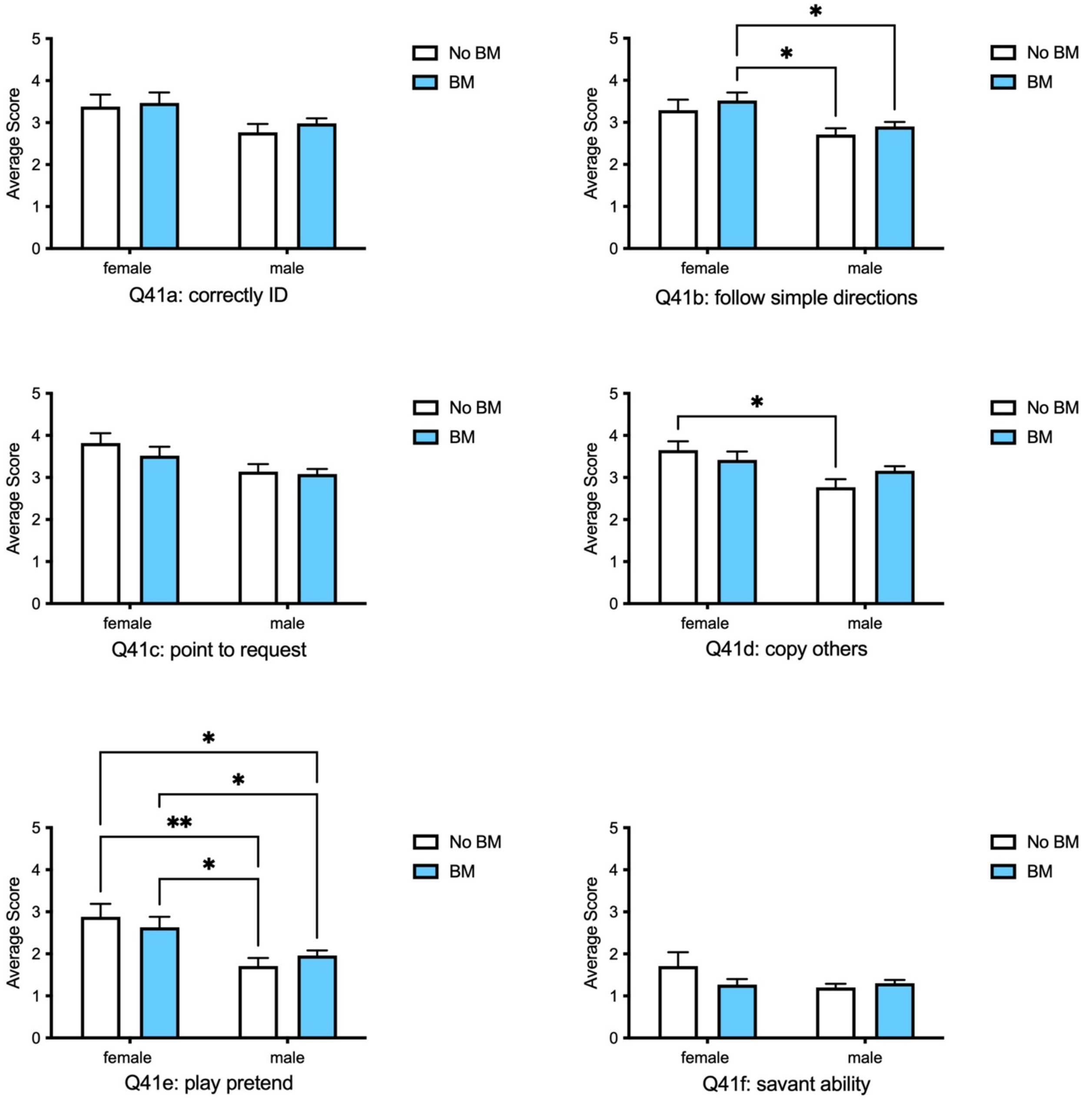

Supplementary Figure S4. Average child play scores as a function of breast milk. The average scores for individual Child Play questions were plotted versus sex where white bars represent no breast milk and blue bars represent plus breast milk. Error bars represent SEM. Asterisks indicate statistical significance by 2-way ANOVA and Tukey posthoc tests as defined as *P<0.05.

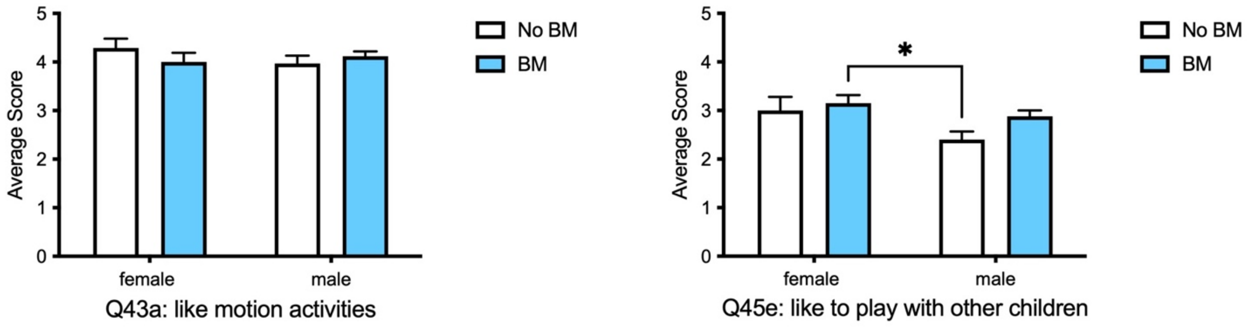

Supplementary Figure S5. Average motor skills scores as a function of breast milk. The average scores for individual Motor Skills questions were plotted versus sex where white bars represent no breast milk and blue bars represent plus breast milk. Error bars represent SEM. Asterisks indicate statistical significance by 2-way ANOVA and Tukey posthoc tests as defined as **P*<0.05.

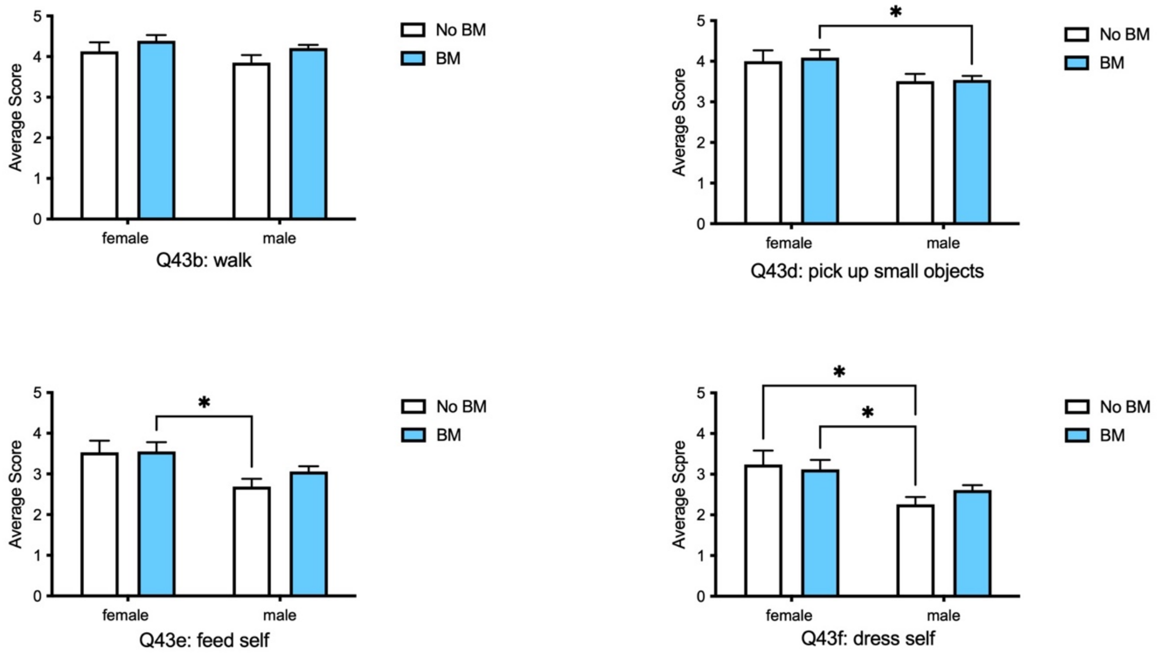

Supplementary Figure S6. Average autism behaviors scores as a function of breast milk. The average scores for individual Autism Behavior questions were plotted versus sex where white bars represent no breast milk and blue bars repre-sent plus breast milk. Error bars represent SEM. Asterisks indicate statistical significance by 2-way ANOVA and Tukey posthoc tests as defined as ****P*<0.001 and *****P*<0.0001.

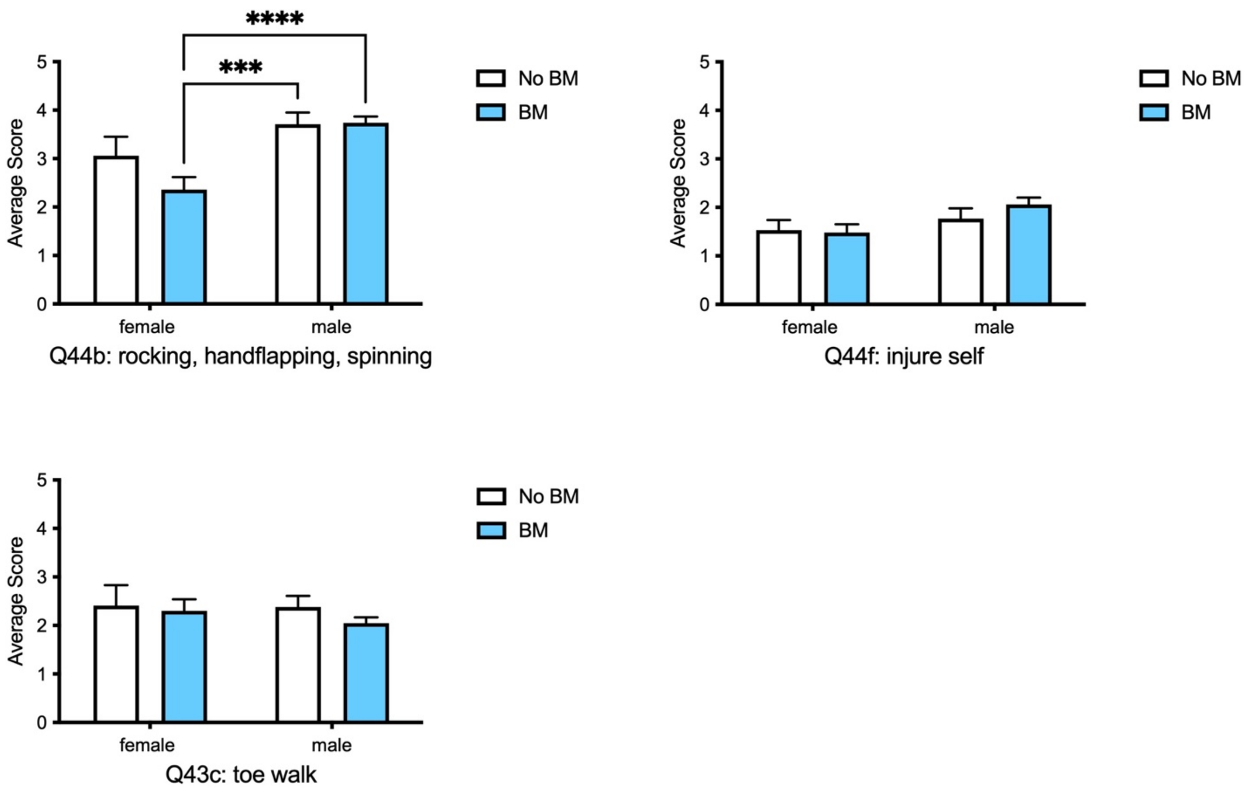

Supplementary Figure S7. Average hypersensory scores as a function of breast milk. The average scores for individual Hypersensory questions were plotted versus sex where white bars represent no breast milk and blue bars represent plus breast milk. Error bars represent SEM.

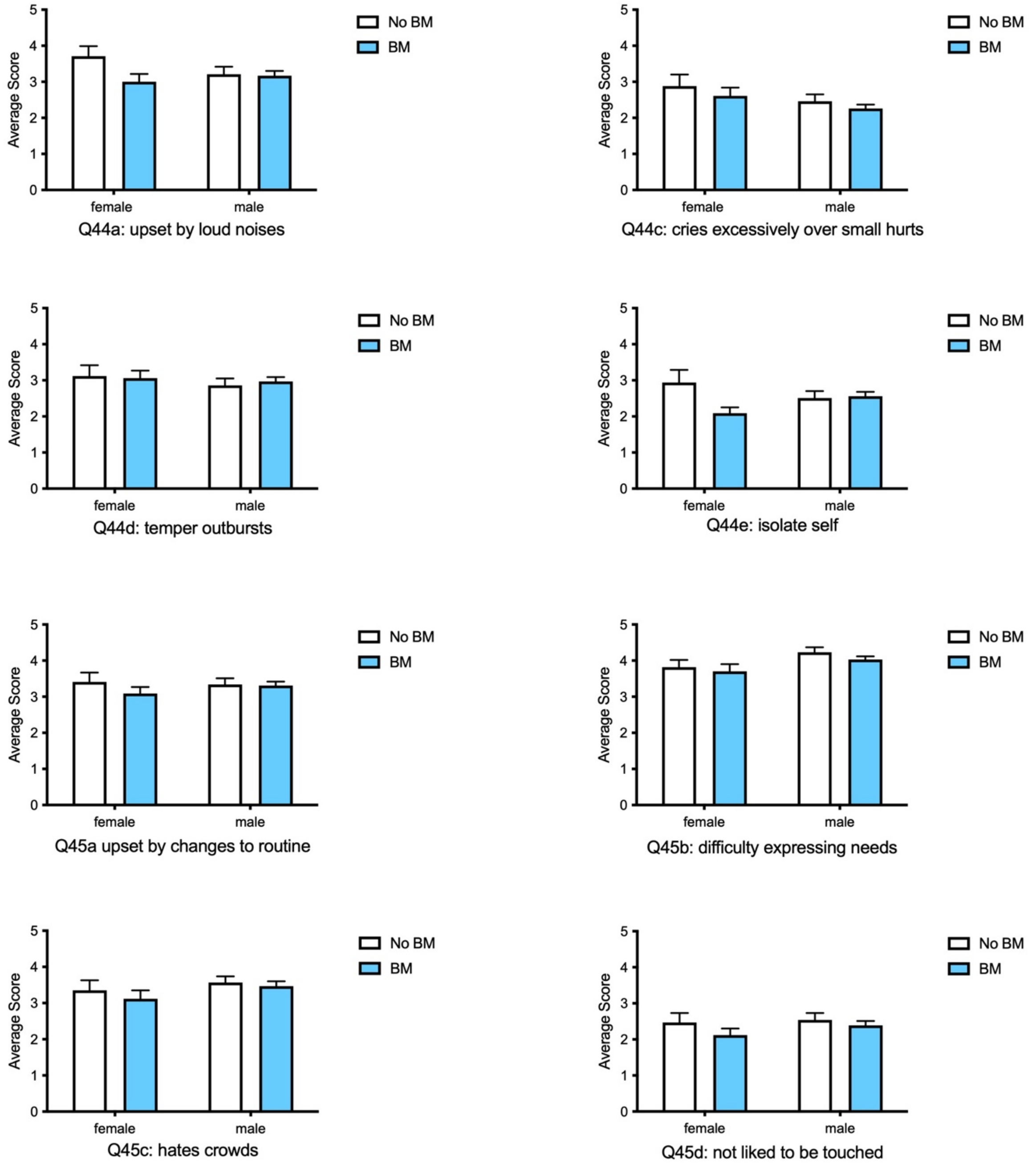

Supplementary Figure S8. Average parents thought there was a problem scores as a function of breast milk. The average scores for individual Parent Thought Problem questions were plotted versus sex where white bars represent no breast milk and blue bars represent plus breast milk. Error bars represent SEM.

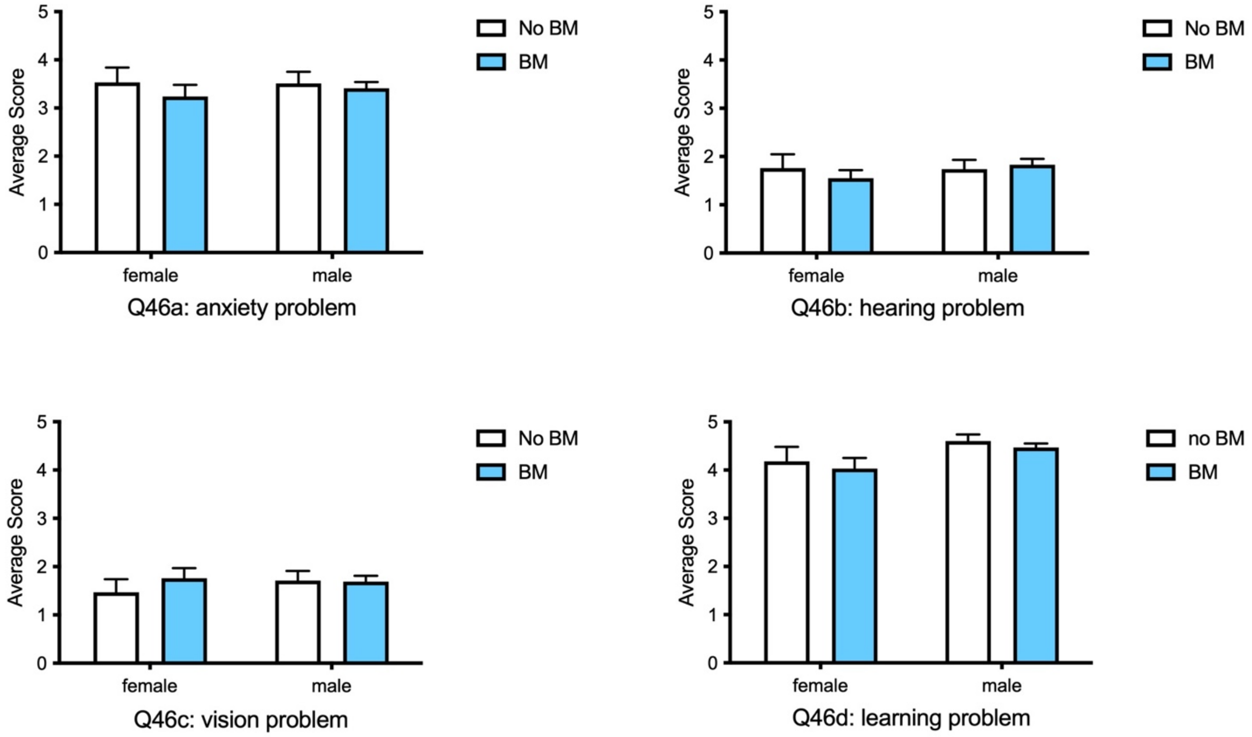

Supplementary Figure S9. Average behavior scores as a function of breast milk for 12 months. The average behavior scores for grouped Language, Cognition, Child Play, Motor Skills, Autistic Behaviors, Hypersensory, and Parent Though Problem questions were plotted versus sex where white bars represent no breast milk and purple bars represent plus breast milk for 12 months. Error bars represent SEM. Asterisks indicate statistical significance by 2-way ANOVA and Tukey posthoc tests as defined as *P<0.05, **P<0.01, and ***P<0.001.

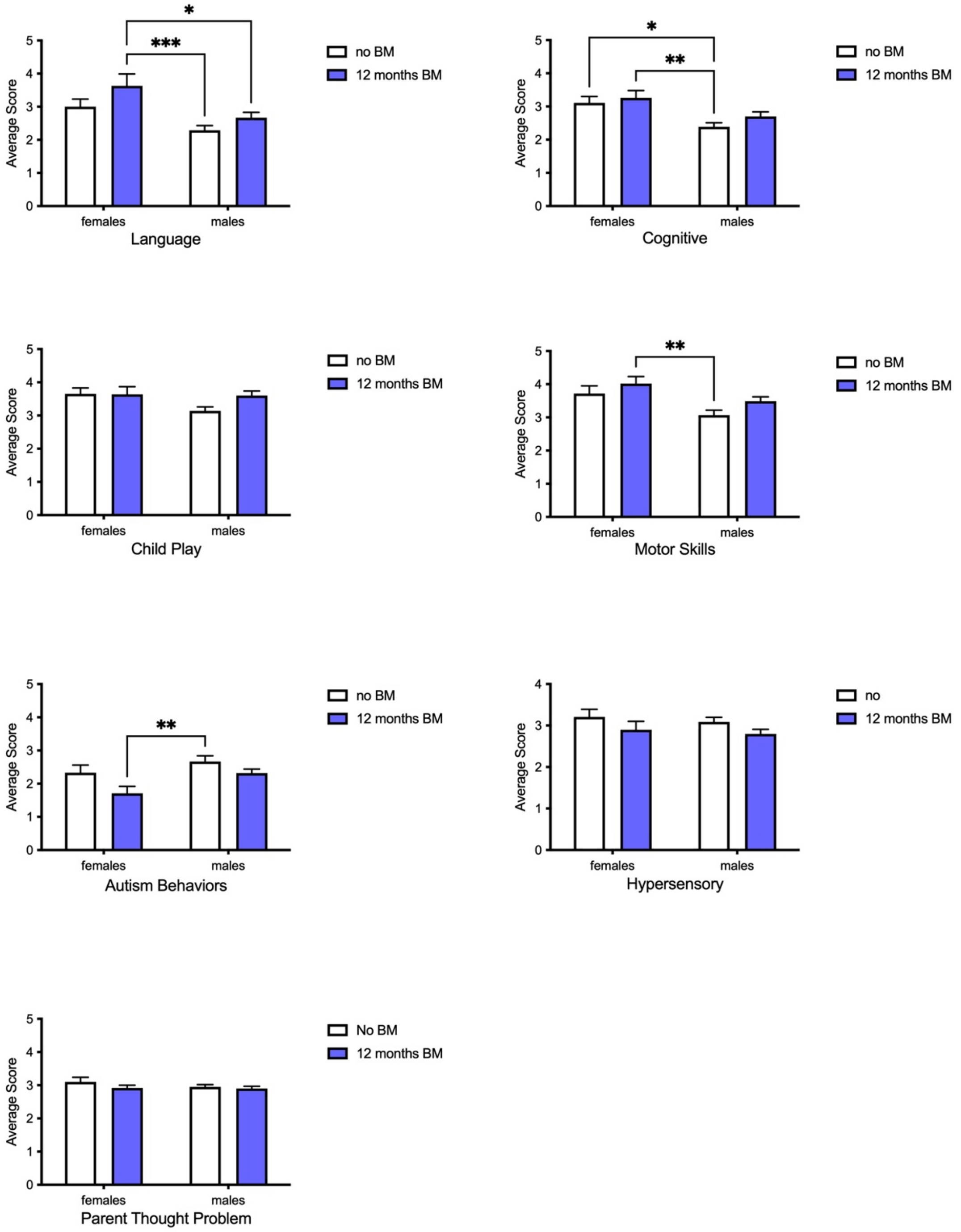

Supplementary Figure S10. Average behavior scores as a function of soy-based infant formula. The average behavior scores for grouped Language, Cognition, Child Play, Motor Skills, Autistic Behaviors, Hypersensory, and Parent Though Problem questions as well as the Total behavior score were plotted versus sex where white bars represent no soy-based infant formula and green bars represent plus soy-based infant formula. Error bars represent SEM. Asterisks indicate statistical significance by 2-way ANOVA and Tukey posthoc tests as defined as *P<0.05, **P<0.01, ***P<0.001, and ****P<0.0001.

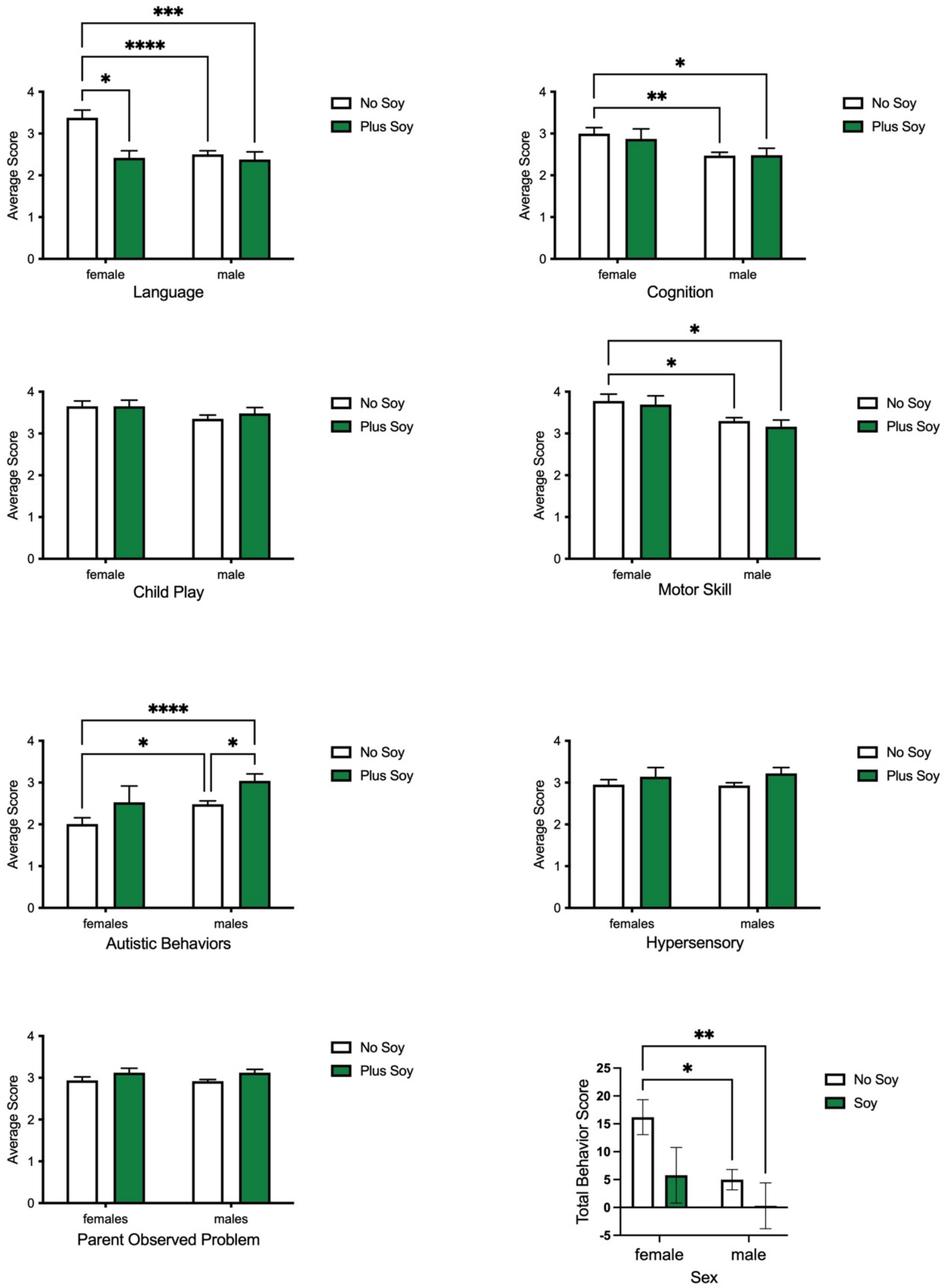

Supplementary Figure S11. Average language scores as a function of soy-based infant formula. The average scores for individual Language questions were plotted versus sex where white bars represent no soy-based infant formula and green bars represent plus soy-based infant formula. Error bars represent SEM. Asterisks indicate statistical significance by 2-way ANOVA and Tukey posthoc tests as defined as *$P<0.05$, **$P<0.01$, ***$P<0.001$, and ****$P<0.0001$.

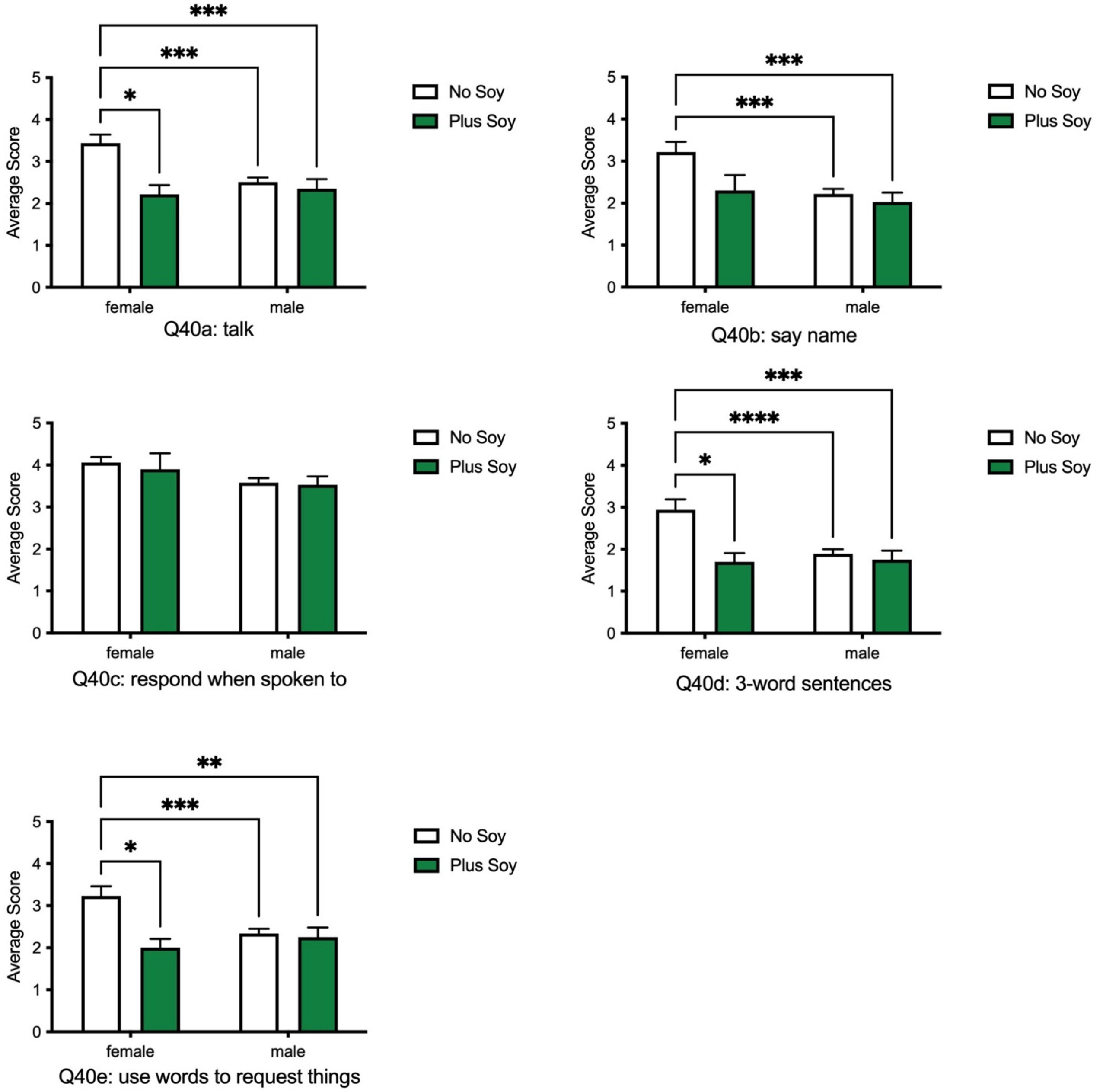

Supplementary Figure S12. Average cognition behaviors scores as a function of soy-based infant formula. The average scores for individual Cognition questions were plotted versus sex where white bars represent no soy-based infant formula and green bars represent plus soy-based infant formula. Error bars represent SEM. Asterisks indicate statistical significance by 2-way ANOVA and Tukey posthoc tests as defined as *P<0.05 and ***P<0.001.

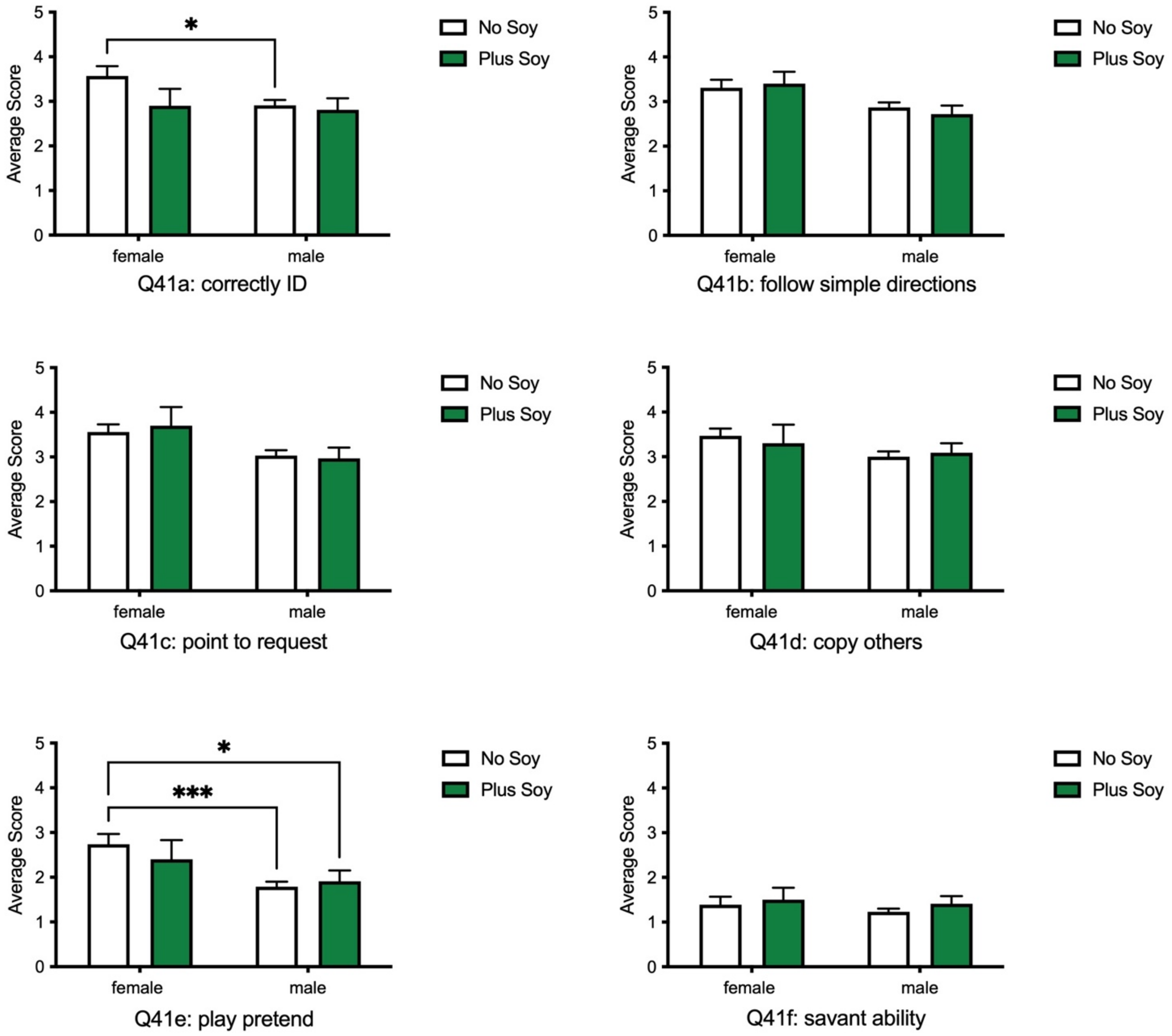

Supplementary Figure S13. Average autism behaviors scores as a function of soy-based infant formula. The average scores for individual Autism Behaviors questions were plotted versus sex where white bars represent no soy-based infant formula and green bars represent plus soy-based infant formula. Error bars represent SEM. Asterisks indicate statistical significance by 2-way ANOVA and Tukey posthoc tests as defined as $^{**}P<0.01$, and $^{****}P<0.0001$.

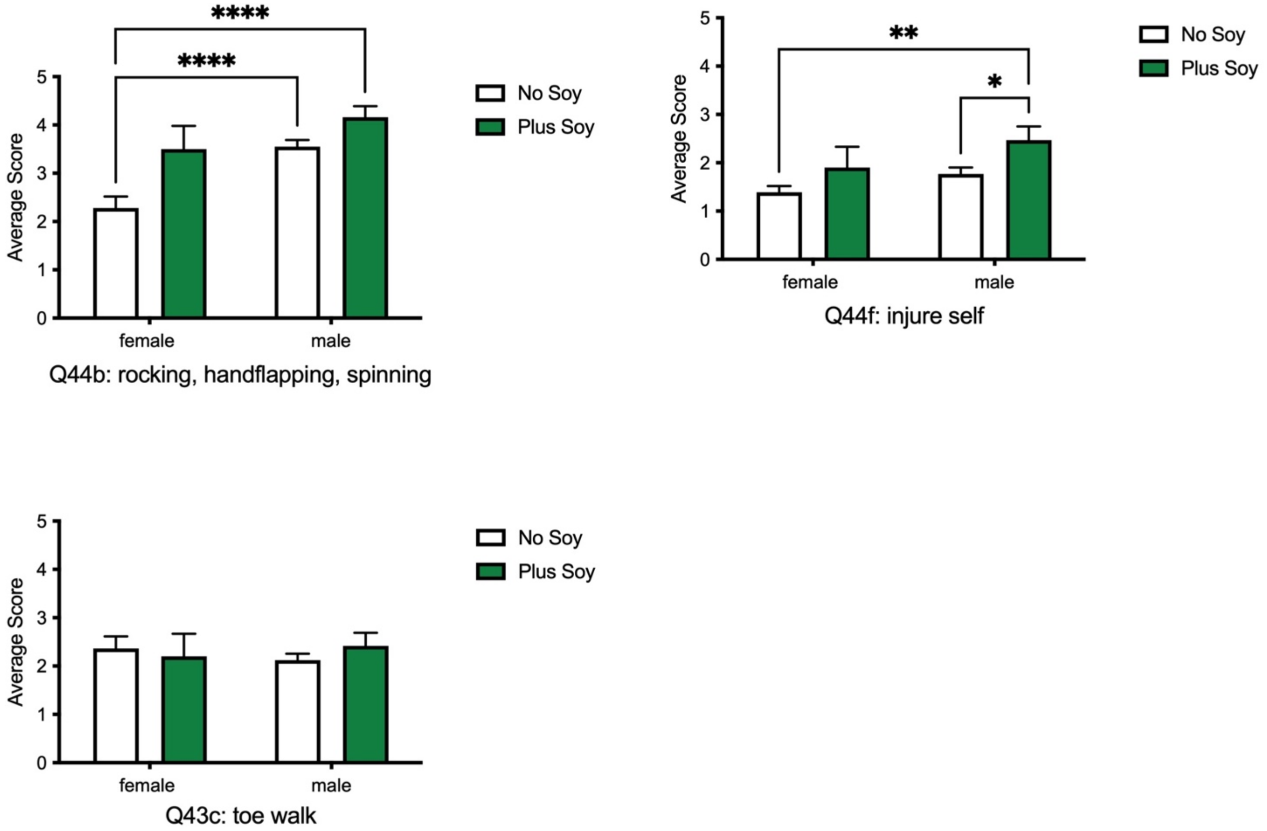

Supplementary Figure S14. Average child play scores as a function of soy-based infant formula. The average scores for individual Child Play questions were plotted versus sex where white bars represent no soy-based infant formula and green bars represent plus soy-based infant formula. Error bars represent SEM.

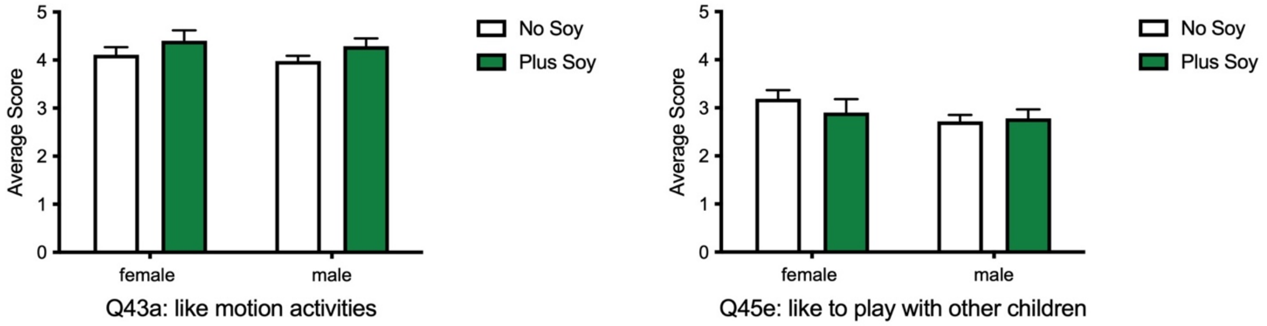

Supplementary Figure S15. Average motor skills scores as a function of soy-based infant formula. The average scores for individual Motor Skills questions were plotted versus sex where white bars represent no soy-based infant formula and green bars represent plus soy-based infant formula. Error bars represent SEM. Asterisks indicate statistical significance by 2-way ANOVA and Tukey posthoc tests as defined as *P<0.05 and **P<0.01.

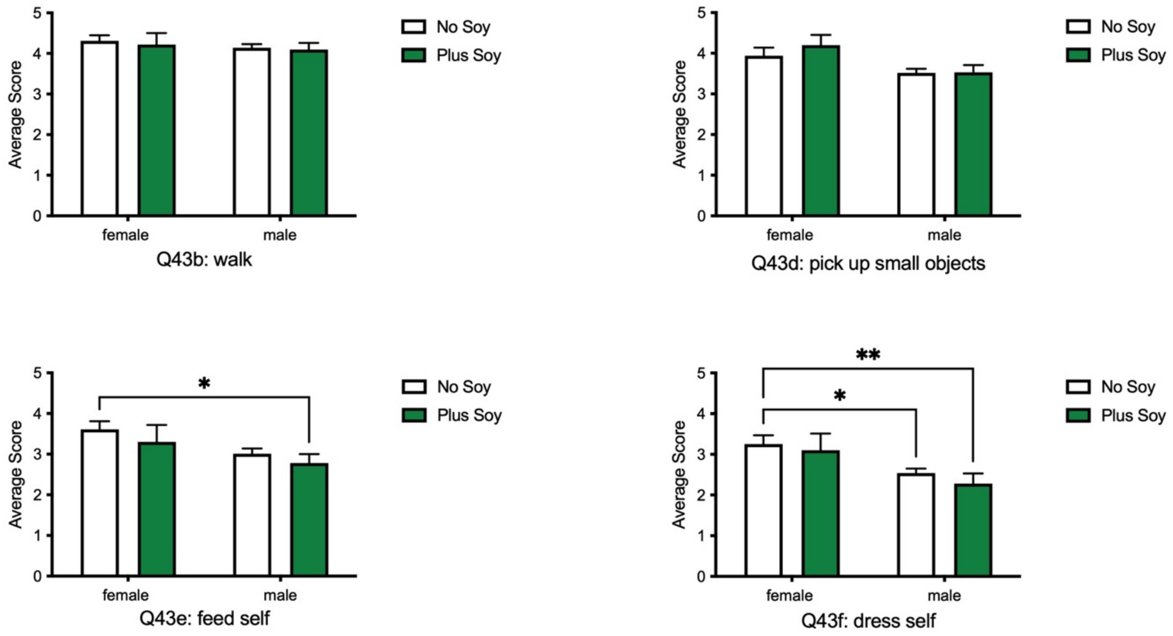

Supplementary Figure S16. Average hypersensory scores as a function of soy-based infant formula. The average scores for individual Hypersensory questions were plotted versus sex where white bars represent no soy-based infant formula and blue bars represent plus soy-based infant formula. Error bars represent SEM. Asterisks indicate statistical significance by 2-way ANOVA and Tukey posthoc tests as defined as $^{**}P<0.01$.

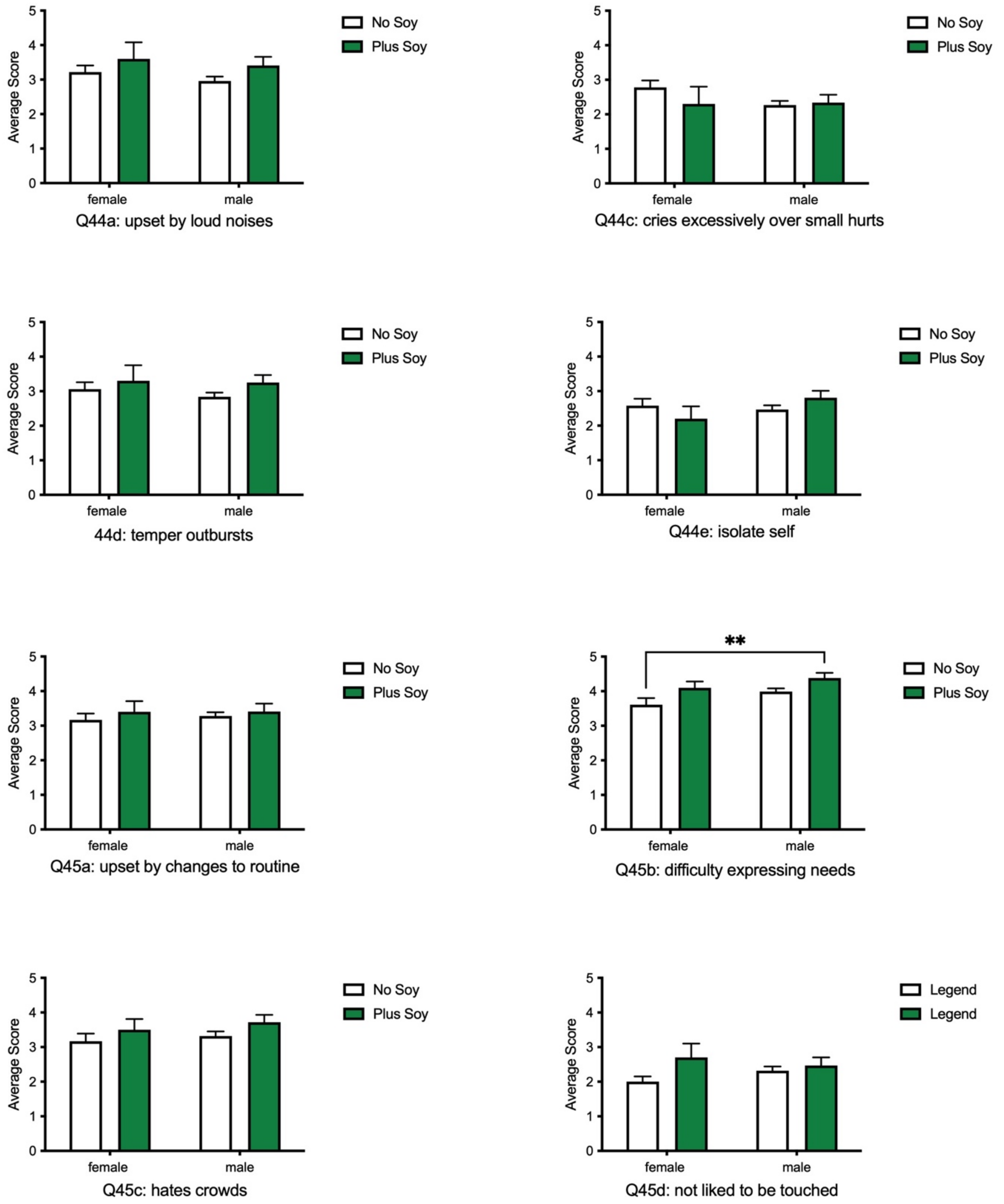

Supplementary Figure S17. Average parents thought there was a problem scores as a function of soy-based infant formula. The average scores for individual Parent Thought Problem questions were plotted versus sex where white bars represent no soy-based infant formula and blue bars represent plus soy-based infant formula. Error bars represent SEM. Asterisks indicate statistical significance by 2-way ANOVA and Tukey posthoc tests as defined as *P<0.05 and **P<0.01.

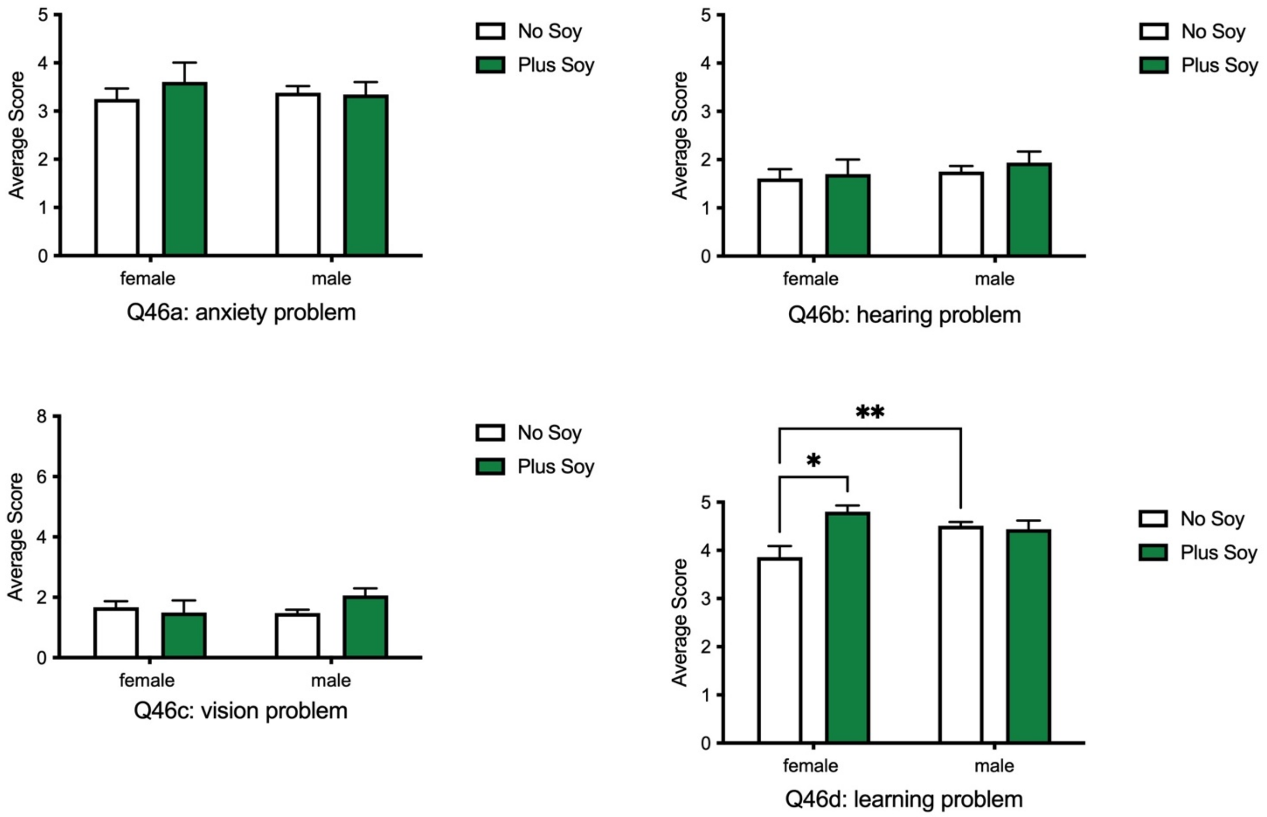

Supplementary Figure S18. Average behavior scores as a function of single milk. The average behavior scores for grouped Language, Cognition, Child Play, Motor Skills, Autistic Behaviors, Hypersensory, and Parent Though Problem questions were plotted versus sex where blue bars represent only breast milk, pink bars represent only cow milk-based formula and green bars represent only soy-based infant formula. Error bars represent SEM. Asterisks indicate statistical significance by 2-way ANOVA and Tukey posthoc tests as defined as *P<0.05.

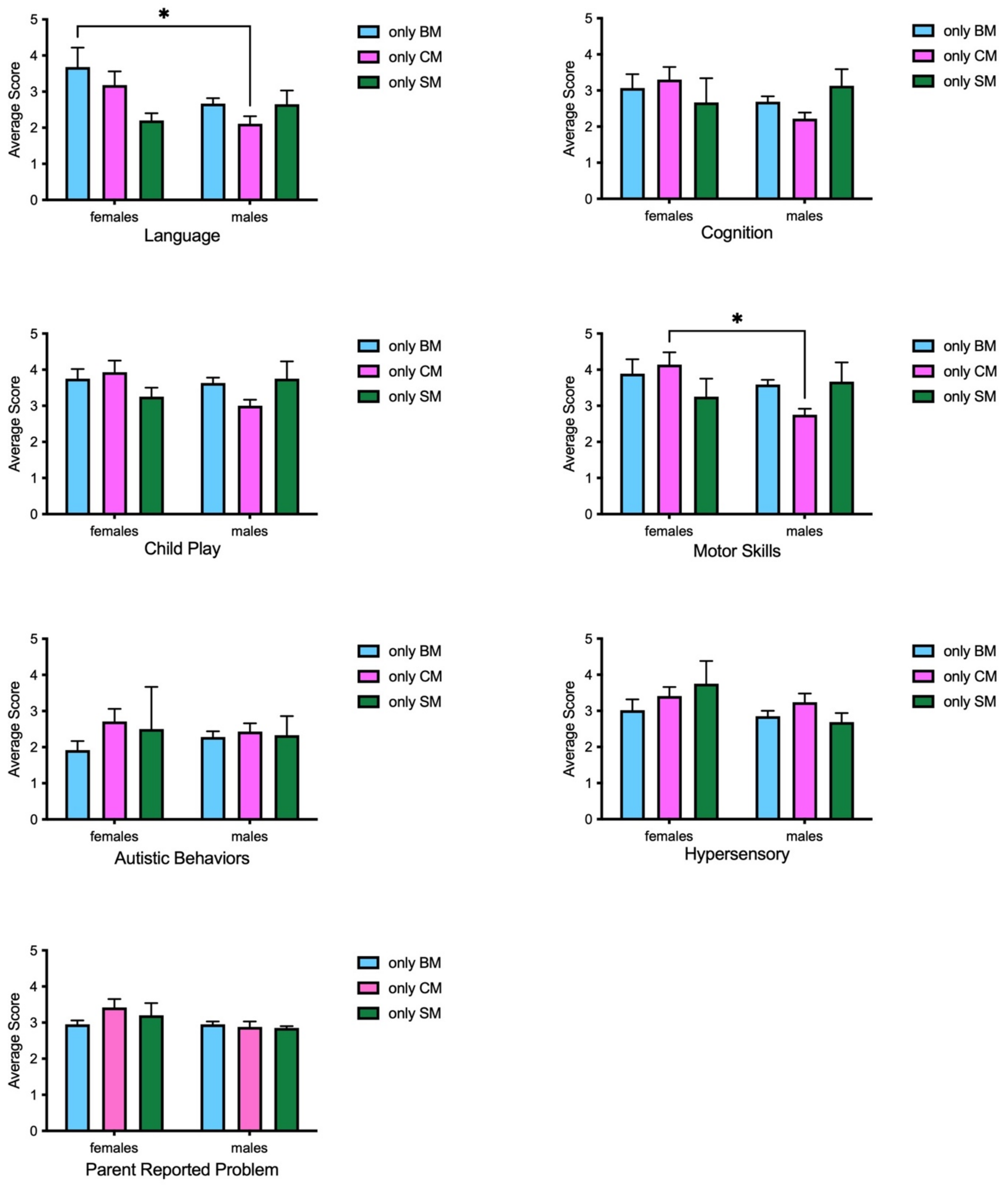

Supplementary Table S1: Average Behavior Category Statistics as a Function of Breast Milk

	Interaction		Sex		BM[a]	
	F[b]	*P*	F[b]	*P*	F[b]	*P*
Language	0.0033	0.95	16.33	<0.0001	2.61	0.11
Cognitive	1.18	0.28	15.71	0.0001	0.011	0.92
Child Play	2.42	0.12	4.55	0.034	1.10	0.30
Motor Skills	0.47	0.49	12.71	0.0005	1.31	0.25
Autistic Behavior	0.43	0.51	7.14	0.0082	1.02	0.31
Hypersensory	1.40	0.24	0.042	0.84	3.07	0.082
Parent Thought Problem	2.52	0.11	0.16	0.69	0.63	0.43
Total	0.075[c]	0.79	9.71[c]	0.0022	3.10[c]	0.080

[a] BM = breast milk. [b] F = 2-way ANOVA F(1,183) statistic except where indicated.
[c] F = 2-way ANOVA (F, 159) statistic.

Supplementary Table S2: Average Behavior Category Statistics as a Function of BM 12 Months

	Interaction		Sex		BM 12 months[a]	
	F[b]	*P*	F[b]	*P*	F[b]	*P*
Language	0.34	0.56	15.18	0.0002	5.55	0.020
Cognitive	0.21	0.65	13.56	0.0004	1.75	0.19
Child Play	1.83	0.18	2.51	0.12	1.68	0.20
Motor Skills	0.11	0.75	10.18	0.0019	3.79	0.054
Autistic Behavior	0.51	0.48	6.31	0.014	6.58	0.012
Hypersensory	0.0045	0.95	0.54	0.46	4.02	0.048
Parent Thought Problem	0.47	0.50	0.80	0.37	1.46	0.23

[a] BM = breast milk 12 months. [b] F = 2-way ANOVA F(1, 103) statistic.

Supplementary Table S3: Average Behavior Category Statistics as a Function of Soy Formula

	Interaction		Sex		SM[a]	
	F[b]	*P*	F[b]	*P*	F[b]	*P*
Language	4.81	0.030	5.77	0.017	7.95	0.0054
Cognitive	0.17	0.68	7.38	0.0073	0.13	0.72
Child Play	0.15	0.70	1.95	0.16	0.15	0.70
Motor Skills	0.021	0.88	8.65	0.0037	0.45	0.50
Autistic Behavior	0.013	0.91	7.55	0.0067	9.17	0.0029
Hypersensory	0.12	0.73	0.042	0.84	2.71	0.10
Parent Thought Problem	0.014	0.91	0.014	0.91	4.88	0.029
Total	0.54[c]	0.46	4.63[c]	0.033	3.78[c]	0.054

[a] SM = soy-based infant formula. [b] F = 2-way ANOVA F(1,166) statistic except where indicated.
[c] F = 2-way ANOVA (F, 147) statistic.

Supplementary Table S4: Average Behavior Category Statistics as a Function of Single Diet

	Interaction		Sex		Single Diet[a]	
	F[b]	*P*	F[c]	*P*	F[b]	*P*
Language	1.47	0.24	2.58	0.11	2.34	0.11
Cognitive	1.80	0.17	1.18	0.28	0.11	0.90
Child Play	2.06	0.14	0.41	0.52	0.43	0.65
Motor Skills	3.39	0.041	2.13	0.15	0.75	0.48
Autistic Behavior	0.70	0.50	0.0089	0.93	1.43	0.25
Hypersensory	0.73	0.48	2.65	0.11	1.23	0.30
Parent Thought Problem	1.85	0.17	3.44	0.069	0.98	0.38

[a] Single Diet = caregivers only reported use of breast milk or cow milk-based formula or soy-based formula. [b] F = 2-way ANOVA $F(2, 56)$ statistic. [c] F = 2-way ANOVA $F(1, 56)$ statistic.
